# Sinking cities, rising seas

**DOI:** 10.1038/s44172-022-00033-4

**Published:** 2022-11-03

**Authors:** Mengying Su

**Affiliations:** Communications Engineering, https://www.nature.com/commseng/

## Abstract

Land subsidence adds to the problem of climate-driven sea-level rise in coastal regions. A recent publication in Nature Sustainability has quantified the relative rates of local land subsidence of 48 major coastal cities worldwide. The study found that relative local land subsidence is more spatially variable than IPCC estimates previously suggested, with cities in Asia suffering the most. The findings could refine predictions of relative sea level rise and better guide actions for planning, designing and implementing protection strategies for coastal cities.

**Figure:**
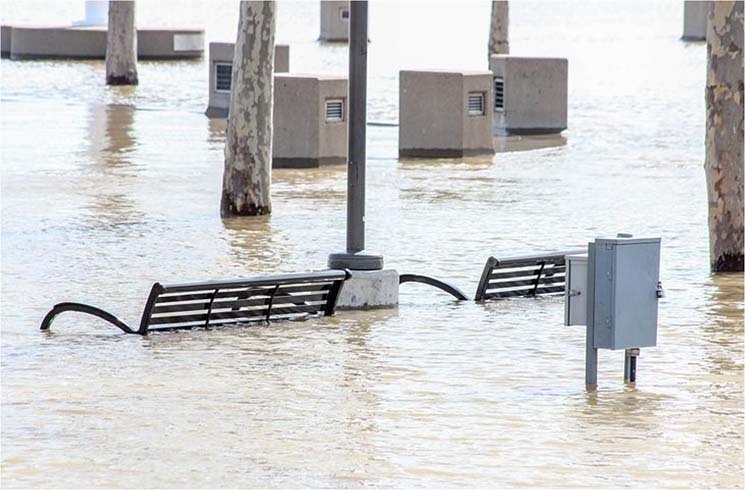
Pixabay

Even a small increase in sea level will pose a great threat to coastal land. Projecting future sea level rise is critical for policymakers, city planners and engineers to develop sustainable adaptation and mitigation strategies. The Sixth Assessment Report of the United Nations Intergovernmental Panel on Climate Change (IPCC AR6) assesses the scientific, technical and socio-economic information regarding the climate change. However, the AR6, alongside with many other studies^1,2^, reports the global relative sea-level rise (RSLR) over large geographical areas, underestimating the influence from local land subsidence^1^.

Remote sensing and earth observation techniques such as interferometric synthetic aperture radar (InSAR) have a major role to play in measuring and projecting local land subsidence. InSAR monitors the ground by comparing the phase change of the microwave radar signals between two aperture radar (SAR) images. InSAR deformation maps could provide continuous monitoring capacity with wide spatial coverage and high resolution in all weathers^2^, making it an effective tool for local land subsidence estimation. However, disparities in processing approaches, datasets, time spans and a loss of coherence over vegetated areas have been barriers to consolidating local land subsidence data from existing studies. Thus vertical land motion has largely been assumed to be similar across localities.

Cheryl Tay and colleagues from Nanyang Technological University used InSAR data from 2014 to 2018 to produce coherent InSAR maps by spatially interpolating the velocities across the vegetation growth or extensive land disturbance areas. They then derived consistent relative local land subsidence velocities for 48 of the world’s largest coastal cities. Different from the vertical land motion reported by the IPCC AR6, the relative local land subsidence velocity reported in this work does not include the broad-scale components, such as regional tectonics and glacial isostatic adjustment. Therefore, the difference between the local and global land subsiding velocities could indicate the contribution from local land subsidence on the global sea-level rise.

Dr. Tay and collaborators compared relative local subsidence velocities across the major coastal cities worldwide and used negative velocity to refer the land subsidence compared to mean sea level. The results showed that the fast-subsiding cities with the highest subsidence velocity of faster than −20 mm per year, are concentrated in Asia, especially those megacities experiencing rapid expansion. 44 out of 48 cities have experienced faster land subsiding than the global mean sea-level rise of −3.7 mm/year reported by IPCC AR 6 between 2006 and 2018. The researchers also observed that local land subsidence velocities are more various across cities (−16.2 mm/year to 1.1 mm/year) than the values estimated by IPCC AR6 (−5.2 to 4.9 mm/year). The authors suggest that the coastal cities are probably exposed to a greater extent of relative sea level rise when adding the contribution from the local land subsidence. Dr. Tay and her colleagues found that high spatial variability even exists within a city related to human activities, such as industrial, agricultural or aquacultural groundwater use.

The methodology and results presented can be applied more broadly to update the current estimation of relative sea-level rise impact to include the influences from local land subsidence. An illustrative example shows that the inundation extents could increase by 20 km^2^ and 2 km^2^ in Ho Chi Minh City (Vietnam) and Rio de Janeiro (Brazil), respectively, by 2030 when accounting for the effect of local land subsidence. The authors also suggest that these site-specific factors should be considered in analyzing more long-term sea level rise.

Costal subsidence over time and space is complex. The researchers point out that overlooking details of local spatial variation will put “cities experiencing rapid local land subsidence at greater risk of coastal hazards than already present due to climate-driven sea-level rise”. Integrating local subsidence information in future sea level rise assessment will “better inform the sustainable planning”.

The original article can be found here: Tay, C., Lindsey, E. O., Chin, S. T. et al. Sea-level rise from land subsidence in major coastal cities. *Nat. Sustain.* 10.1038/s41893-022-00947-z (2022).
